# The Subcellular Localization and Functional Analysis of Fibrillarin2, a Nucleolar Protein in* Nicotiana benthamiana*

**DOI:** 10.1155/2016/2831287

**Published:** 2016-01-17

**Authors:** Luping Zheng, Jinai Yao, Fangluan Gao, Lin Chen, Chao Zhang, Lingli Lian, Liyan Xie, Zujian Wu, Lianhui Xie

**Affiliations:** ^1^Key Laboratory of Biopesticide and Chemical Biology, Ministry of Education, Fujian Agriculture and Forestry University, Fuzhou 350002, China; ^2^Key Laboratory of Plant Virology of Fujian Province, Fujian Agriculture and Forestry University, Fuzhou 350002, China; ^3^Institute of Plant Protection, Fujian Provincial Academy of Agricultural Sciences, Fuzhou 350002, China

## Abstract

Nucleolar proteins play important roles in plant cytology, growth, and development. Fibrillarin2 is a nucleolar protein of* Nicotiana benthamiana *(*N. benthamiana*). Its cDNA was amplified by RT-PCR and inserted into expression vector pEarley101 labeled with yellow fluorescent protein (YFP). The fusion protein was localized in the nucleolus and Cajal body of leaf epidermal cells of* N. benthamiana*. The* N. benthamiana* fibrillarin2 (NbFib2) protein has three functional domains (i.e., glycine and arginine rich domain, RNA-binding domain, and *α*-helical domain) and a nuclear localization signal (NLS) in C-terminal. The protein 3D structure analysis predicted that NbFib2 is an *α*/*β* protein. In addition, the virus induced gene silencing (VIGS) approach was used to determine the function of NbFib2. Our results showed that symptoms including growth retardation, organ deformation, chlorosis, and necrosis appeared in NbFib2-silenced* N. benthamiana.*

## 1. Introduction

Fibrillarin is a major nucleolar protein, playing multifunctional roles in RNA biogenesis. It localizes in nucleolus and Cajal bodies (CBs), subnuclear dynamic particles involved in RNA transcription and editing [1]. Fibrillarin is an evolutionarily conserved protein. Human fibrillarin shares 94.2% sequence identity with mouse fibrillarin and 82.9% sequence identity with amphibian fibrillarin [2]. Homologs of human fibrillarin are also reported in advanced plants [3].

Fibrillarin usually consists of three domains including a glycine and arginine rich domain (GAR domain), an RNA-binding domain, and an *α*-helical domain. The GAR domain is critical for the function of fibrillarin. This domain is localized in the N-terminal of the protein and its arginine residues are methylated [4]. In human and plants, the GAR domain is involved in the translocation of fibrillarin into nucleoli. The RNA-binding domain, which interacts with RNA [5] and *α*-helical domain, which interacts with nucleolar protein 56 (Nop56) [6], is localized in the middle and C-terminal of fibrillarin, respectively. The RNA-binding domain and *α*-helical domain together form an Ado-Met-dependent methyltransferase- (MTase-) like region. The MTase-like region, which is evolutionarily conserved, contains S-adenosylmethionine (SAM, the methionine group donor) binding motifs and encodes a MTase required for the methylation of 2′-O-ribose [5].

In addition to understanding its functions in pre-rRNA processing, modification, and ribosomal assembly [7], researches in fibrillarin are focused on its interactions with virus-encoded-proteins and the roles of these interactions in viral movement and infection. For example, Melén et al. found that fibrillarin interacted with nonstructural protein 1 (NS1) in* Influenza A *H3N2 subtype virus via C-terminal nuclear localization signal 2 (NLS2) [8]. Kim et al. found that fibrillarin is involved in the long distance movement and infection of* Groundnut rosette virus *(GRV) [9, 10]. Specifically, ORF3 (movement protein) in GRV migrates into nucleolus via CBs, binds with fibrillarin in nucleolus, relocates into cytoplasm, and finally assembles with virus ribonucleoprotein (vRNP) particles in cytoplasm for long-distance movement and systemic infection. It was also reported that fibrillarin interacts with viral genome-linked protein (VPg) in* Potato virus A *(PVA) and 2b silencing suppressor protein in* Cucumber mosaic virus *(CMV) [11, 12].

It is known that genes in some plants can be cosuppressed if the plants are transformed with homologous transgenes. This mechanism will result in new types of intercellular communication and viral defense mechanisms [13]. The event in which virus vectors carrying the host-derived sequence silence the corresponding host genes in the infected plants is defined as virus-induced gene silencing (VIGS) [14]. VIGS is the manifestation of an RNA-mediated defense mechanism and is believed to be a fast and powerful method to determine gene function. In 2001, a novel VIGS vector TRV was modified from the RNA virus* Tobacco rattle virus*. This vector successfully silenced endogenous genes such as* phytoene desaturase* (*PDS*) and* Nicotiana FLO/LEY* (*NFL*) in* Nicotiana benthamiana* (*N. benthamiana*) [14]. Since then, TRV has become the most commonly used vector in VIGS studies. The vector used in this study is a modified TRV [15], containing duplicated CaM V 35S promoters (2 × 35S) and nopaline synthase terminator (NOSt) in the C-terminal, which can ensure the accumulation of viral RNAs to a higher level. The new TRV has two genomes, designated as pTRV1 and pTRV2 (pYL156). The latter genome acts as a VIGS vector containing multiple cloning sites.

Fibrillarin2 is a protein in Fibrillarin family. In our previous study, we identified the importance of fibrillarin2 from* N. benthamiana *(NbFib2) during the process of* Rice stripe virus* infection [16]. However, the subcellular localization, the 3D structure, and functions of NbFib2 were not fully resolved. The objectives of this study include (1) determining the subcellular localization of fibrillarin2 in* N. benthamiana *(NbFib2); (2) predicting the functional domains and 3 dimensional (3D) structure of NbFib2; and (3) identifying the roles of NbFib2 in plant growth and development.

## 2. Results

### 2.1. The Functional Domains of NbFib2

**Figure 1:**
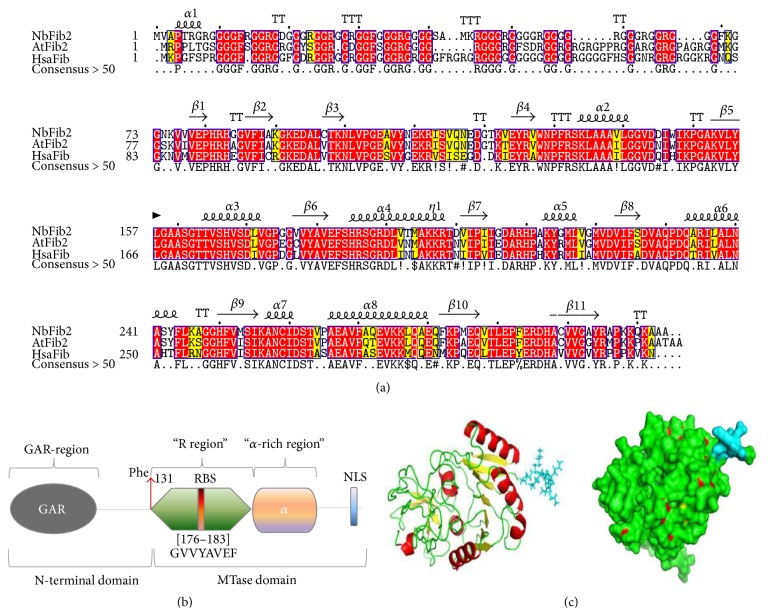
The structural and functional characteristics of NbFib2. (a) Amino acid sequence alignment among NbFib2, AtFib2, and HsaFib; (b) a sketch of functional domains in NbFib2; (c) 3D model of NbFib2 in which the *α*-helical is in red, the *β*-sheet region is in yellow, random coil region is in green, and NLS is in cyan.

NbFib2 is highly homologous to AtFib2 (GenBank accession AAG10153) and HsaFib (GenBank accession AAH19260). They share more than 74% amino acid sequence identity (Figure 1(a)). The protein has 314 amino acid (aa) residues and consists of three functional regions including a GAR region, an RNA binding region, and an *α*-helical region (Figure 1(b)). The GAR region has 61 aas (aa8–68) and is located in the N-terminal of the protein. The RNA binding region is seated in the middle of the protein (aa131–221), in which the most possible interaction sites with RNA are from aa176 to aa183. The *α*-helical region is located near to the C-terminal (aa231–279), followed by a nuclear localization signal (NLS, aa307–313) motif. It is worth mentioning that proline encoded by aa131 is predicted to be involved in most protein activities of NbFib2. The RNA binding region, *α*-helical region, and NLS together form a MTase domain, implying that NbFib2 will localize in nucleus.

In addition, the web server I-TASSER [17] was adopted to predict the 3D structure of NbFib2 using PDB ID 1g8sA (*Pyrococcus horikoshii* fibrillarin) as template (Figure 1(c)). The analysis predicted that NbFib2 belongs to *α*/*β* proteins, a class of structural domains in which the secondary structure is composed of alternating *α*-helices and *β*-sheets along the backbone. The *β*-sheet is an external structure while most part of *α*-helices is internal. The GAR region mostly consists of hairpin-*β* motif. The RNA binding region forms a *β*-*α*-*β* motif and is randomly coiled (Figure 1(c)). The C-terminal region is *α*-helical and the subcellular localization signal peptide is exposed outside.

Furthermore, the quality and reliability of protein structure prediction were evaluated by several assessment methods including the C-score, TM-score, and root-mean-square deviation (RMSD). A high C-score indicates a high confidence in prediction and vice versa, and a protein structure prediction is considered as reliable if its C-score is in the range between −5 and 2. Similarly, the TM-score and RMSD are often used to measure the accuracy of a structure modeling if a reference structure is known. For NbFib2, the C-score of the predicted structure is −2.34, and the estimated TM-score and RMSD are 0.44 ± 0.14 and 11.8 ± 4.5 Å, respectively. These results suggest that the predicted structure of NbFib2 is reliable and closely matched to the topology of the reference protein (e.g., PDB ID 1g8sA).

We then used the COFACTOR server [18] to predict the functions of NbFib2, including the Enzyme Classification (EC) number, Gene Ontology (GO), and protein-ligand binding sites, using the protein structure (PDB ID: 2ipxA) as template. The analysis found that NbFib2 and human fibrillarin share high similarity in ligand-binding sites with an EC value of 0.437, which is in the range of reliable scores for the EC prediction. Thus, the NbFib2 protein might also have a similar active site at residue 131 like human fibrillarin. The function prediction also revealed that NbFib2 has a NLS and several regions targeting nucleolus and Cajal body, suggesting that the protein may localize in the two organelles. These predictions were confirmed by DAPI staining.

### 2.2. Subcellular Localization of NbFib2 and Western Blot Analysis

**Figure 2:**
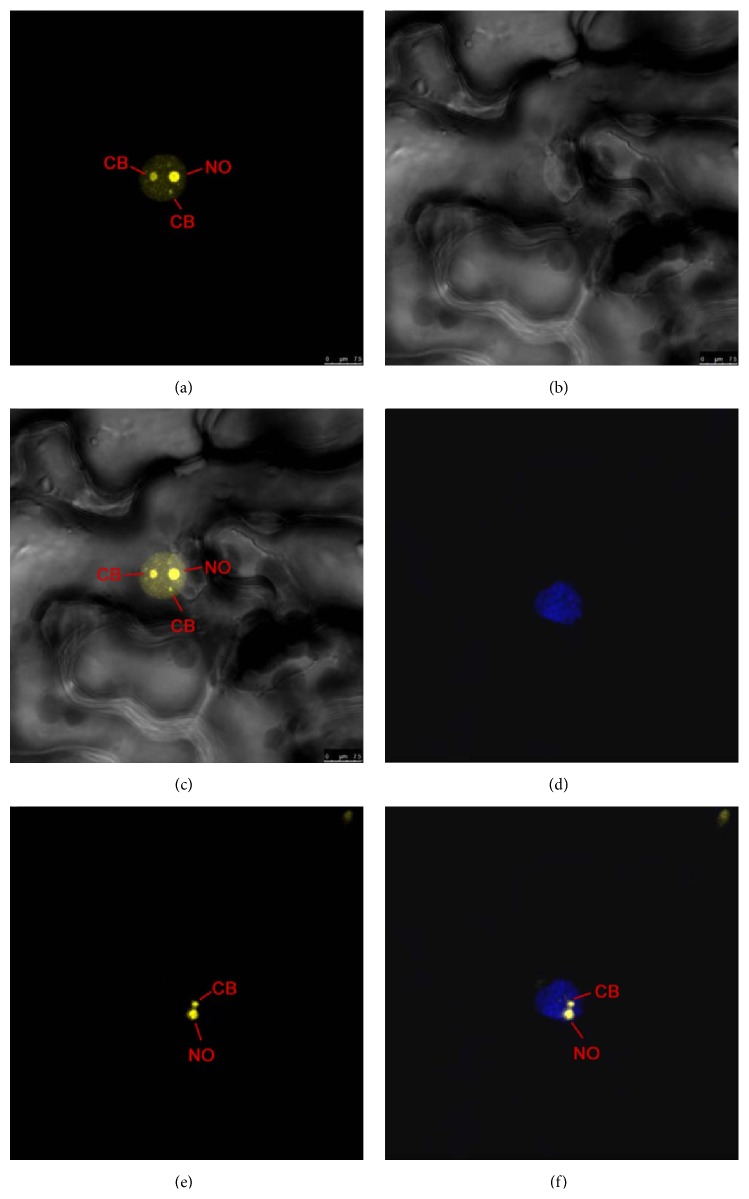
Subcellular localization of pEarley101-NbFib2. (a) pEarley101-NbFib2; (b) bright field; (c) overlay of (a) and (b); (d) DAPI fluorescence staining; (e) pEarley101-NbFib2; (f) overlay of (d) and (e).

**Figure 3:**
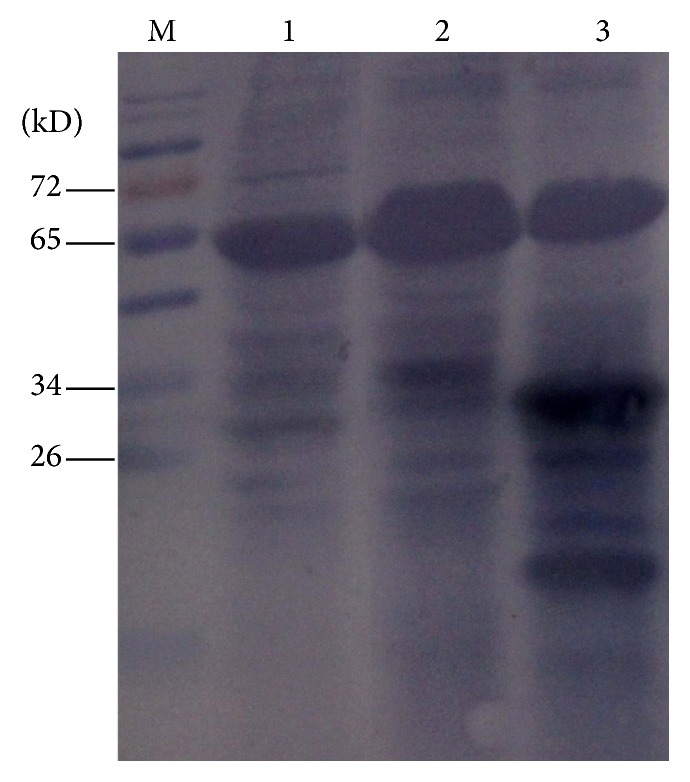
Western blot analysis of proteins expression in* Nicotiana benthamiana* leaves. M: protein Marker; 1: pEarley101-NbFib2; 2: CK (healthy plant); 3: 35S-GFP.

NbFib2 was found in the nucleolus and Cajal body of* N. benthamiana* epidermis cells by DAPI fluorescence staining (Figure 2). Western blot analysis was used to confirm the protein expression. In the leaves inoculated with* Agrobacterium* carrying 35S-GFP, a protein with a molecular weight identical to GFP (30 kD) was detected, while, in the leaves inoculated with* Agrobacterium* carrying pEarley101-NbFib2, a protein with a molecular weight identical to NbFib2-YFP fusion protein (75 kD) was detected. The two proteins were not detected in the negative control leaves (Figure 3).

### 2.3. Verifying the Function of* NbFib2* Using VIGS

**Figure 4:**
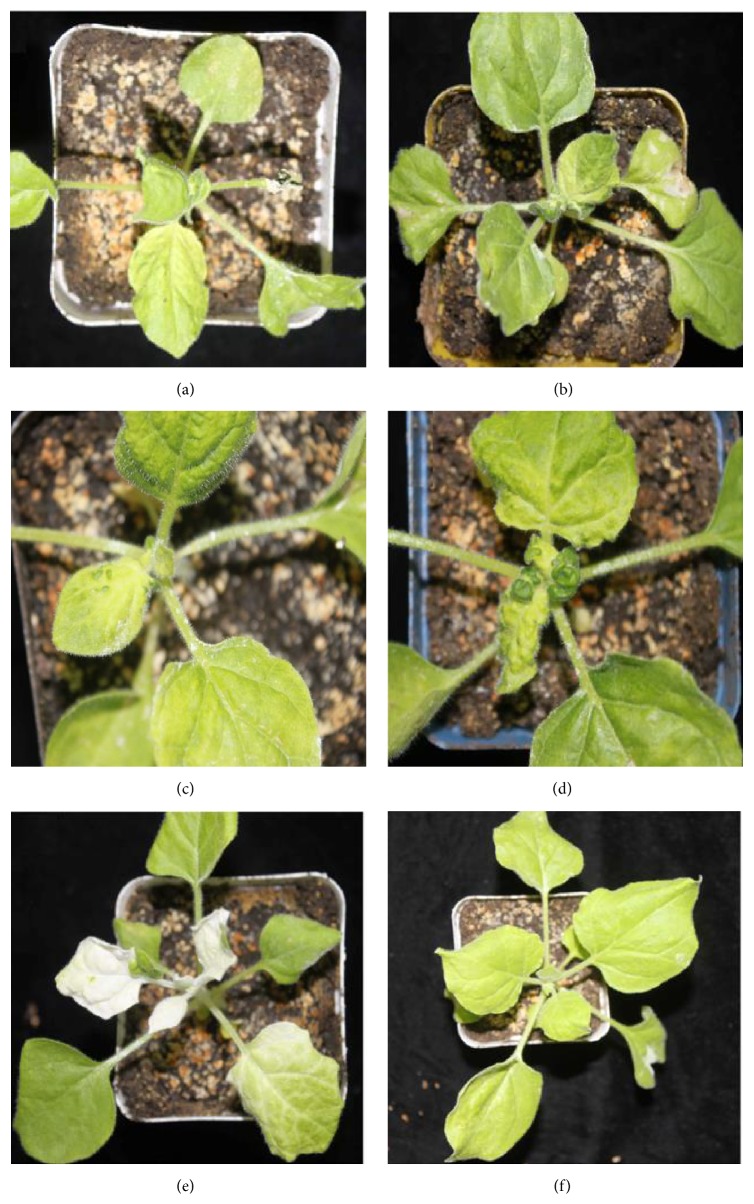
Symptoms of* NbFib2* silenced* Nicotiana benthamiana*. (a)–(d)* NbFib2 *silenced; (e)* NbPDS* (positive control); (f) empty pYL156 (negative control).

Ten days after infiltrating, plants with different genes silenced started to develop different phenotypes. At about three weeks postinoculation (dpi), the obvious and typical symptoms appeared. The leaves in the* NbPDS*-silenced plants (positive control, Figure 4(e)) became bleach, whereas the* NbFib2*-silenced plants developed curved interior leaves (Figures 4(a), 4(b), and 4(d)), chlorotic full leaves (Figures 4(b), 4(c), and 4(d)), and short stems and internodes and became stunted (Figure 4(d)). However, the plants infiltrated by empty TRV vector (negative control, Figure 4(e)) did not develop any symptom.

### 2.4. Semiquantitative RT-PCR Analysis of* NbFib2* mRNA Accumulation

**Figure 5:**
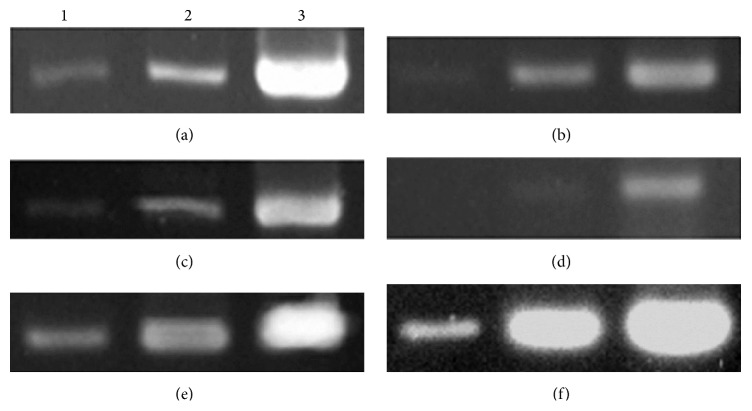
RT-PCR analysis for the accumulation of* NbFib2* transcript in* Nicotiana benthamiana *plants. 1–3: PCR amplifications after 30, 35, and 40 cycles; (a)–(d) amplifications of* NbFib2* from corresponding plants shown in Figures 4(a)–4(d); (e)-(f) amplifications of* NbFib2* and* Nbactin* from plant in Figure 4(f).

The accumulation of* NbFib2 *transcripts in the silenced plants is significantly lower than that in the plants inoculated by empty TRV vector. A negative correlation was shown between transcript level of target genes and the severity of disease symptom; in other words, silenced plants with lower transcript in target genes usually showed more severe symptoms (Figure 5).

## 3. Discussion

NbFib2 shares high homology (more than 74% aa sequence) with AtFib2 and HsaFib; they also contain three functional domains: a GAR domain, an RNA binding domain, and an *α*-helix domain (Figure 1(a)). This result confirmed that NbFib2 belong to fibrillarin protein family, a highly conserved protein family. Previous literatures suggested that the GAR domain always contains a nucleolar localization signal and a targeting site of fibrillarin in cells [19, 20]. In addition, hordeiviral movement protein encoded by the first gene of the triple-gene block (*TGBp1*) in* Poa semilatent virus *(PLSV) interacts with AtFib, which occurs between the GAR domain of AtFib and the N-terminal of TGBp1 [21]. Thus, GAR region might be a potential domain for NbFib2 to bind to virus-encoded proteins. The RNA-binding domain is essential for the presence of fibrillarin in nucleoli [5]. The C-terminal region of Fib protein family always contains two short sequences: one sequence forms an *α*-helix structure, which targets fibrillarin to CBs [1] and interacts directly with Nop56 [6]; the other is NLS. The feature of those domains reveals that NbFib2 should localize in cell nucleus, which is consistent with the results of subcellular localization of NbFib2. The RNA-binding region and C-terminal region can constitute a conserved methyltransferase- (MTase-) like domain, responsible for the methyltransferase activity of fibrillarin [22].

3D structure is very important in determining the functions and localizations of proteins and their interaction with other molecules. Homology modeling is one of the most popular approaches to predict the 3D structure of proteins. Homology modeling requires the identification of a template sequence that matches best the query sequence. The template could be identified using homology search programs such as PSI BLAST against a PDB database using consecutive or spaced seed techniques [23, 24].

The COFACTOR server [18] provides a variety of annotations for functional prediction of proteins. One of the most important advantages of this algorithm is the combination of the global and local structural comparisons. In addition, since COFACTOR takes into account for the global structure similarity, it is more robust than those methods relying on only local pocket comparisons.

VIGS has emerged as a powerful method to study gene functions. In this study, we used VIGS to identify the function of* NbFib2* in the growth and development of* N. benthamiana*. We found that* NbFib2*-silenced* N. benthamiana* plants developed growth retardation, organ deformation, and necrosis. The silenced plants can be divided into two groups based on the reduction of* NbFib2* expression. The plants in the first group developed curly, chlorotic, and deformed interior and upper leaves, while those in the second group developed deformed upper leaves developed shortened stems and internodes and became stunted. No symptoms were developed on the negative control (TRV-empty). We believe that the symptoms of* NbFib2* infiltrated plants are caused by the inserted sequences carried by TRV vector rather than TRV vector itself. The RT-PCR result confirms that the difference in symptom development is consistent with the level of* NbFib2* silenced, suggesting that* NbFib2* contributes to the growth and development of plants in* N. benthamiana*. Fibrillarin is a catalytic component of box C/D small nucleolar ribonucleoproteins (snoRNPs) [25], which is an essential type of proteins for plant growth [26, 27]; fibrillarin also plays important roles on biogenesis of different RNAs and ribosomal subunits; those show that NbFib2 is involved in plant growth and development.

An important feature of plant viral proteins is to interact with fibrillarin, and this feature is not restricted to one or two taxonomic groups [28]. Plant viruses are able to recruit fibrillarin to facilitate their infections in various stages of host development, which suppresses host defense responses. Since* N. benthamiana* is a model plant and the host of many plant viruses, the study of the functions, genetics, and 3D structure of* NbFib2* in* N. benthamiana* is important to understand the interaction between hosts and viruses.

## 4. Materials and Methods

### 4.1. Plant Growth Conditions

The* N. benthamiana* plants were grown and maintained in a greenhouse at 25°C.

### 4.2. Plasmid Construction

Total RNA was extracted from* N. benthamiana* leaves using EasyPure Plant RNA Kit manufactured by Beijing Transgen Biotech Co. Ltd. (Beijing, China). Reverse transcription was carried out using FastQuant RT Kit with gDNase (Tiangen biotech Co., Ltd., Beijing, China). cDNA encoding* NbFib*2 were amplified by PCR using primers (Table 1) designed from* N. benthamiana* sequences (GenBank accession: AM269909) downloaded from GenBank. pEarley101-NbFib2 construct was generated by first inserting* NbFib*2 cDNA into entry vector pDONR221 and then inserting it into destination vector pEarley101 using Gateway recombination system [29]. The constructs were confirmed by capillary sequencing conducted by Takara Biotechnology Co., Ltd. (Dalian, China). The PCR product of* NbFib2* was digested with* Eco*RI and* Bam*HI and ligated into TRV vector by pYL156 digested with the same enzymes.

### 4.3. Functional Domain and 3D Structure Prediction of NbFib2

Fibrillarin sequences from three species were aligned using the MAFFT program (http://mafft.cbrc.jp/alignment/software). Subcellular localization of NbFib2 was predicted by web-based program WolfPsort (http://wolfpsort.org/). Homology identification was performed by submitting NbFib2 sequences into I-TASSR server [17] and the 3D structure of NbFib2 was constructed using* Pyrococcus horikoshii* fibrillarin as reference on the same server. The NbFib2 function was predicted using the COFACTOER server [18].

### 4.4. *Agrobacterium-Mediated* Transient Expression

*Agrobacterium tumefaciens* (*A. tumefaciens*) strain EHA105 carrying pEarley101-NbFib2 and strain GV3101 carrying pTRV1, pYL156-*NbPDS*, and pYL156-*NbFib2 *were grown separately to OD_600_ = 0.8 at 28°C on Luria-Bertani liquid medium supplemented with 50 *μ*g/*μ*L of rifampicin and 50 *μ*g/*μ*L of kanamycin. They were then transferred to induction media (10 mM MES, pH 5.6, 10 mM MgCl_2_, and 150 *μ*M Acetosyringone). The induction media with the bacterial cultures were incubated at room temperature for 3 hours.

Subcellular localization of NbFib2 was determined by infiltrating culture of* A. tumefaciens* harboring pEarley101-NbFib2 onto the fully grown upper leaves of* N. benthamiana*. In the VIGS assay,* A. tumefaciens* harboring pTRV1 was mixed in an equal proportion of* A. tumefaciens* harboring pYL156-*NbFib2*, pYL156-*NbPDS*, and empty pYL156, respectively (V/V), and infiltrated onto fully grown upper leaves. Six-week-old* N. benthamiana* was used for the experiment.

### 4.5. Confocal Imaging Analysis

Subcellular locations of proteins were monitored at 48 hours after infiltration under a confocal microscope (Microsystems CMS GmbH Leica TCS SP5). The fluorophores in YFP were excited using 514 nm light and images were taken using BA535–565-nm emission filters. To locate the fluorescent proteins in nuclei, the* N. benthamiana* leaves were infiltrated with PBS containing 4′,6′-diamidino-2-phenylindole (DAPI).

### 4.6. Western Blot Analysis

Leaves infiltrated with pEarley101-NbFib2 (500 mg) were collected at 72 hours after inoculations and homogenized in 2 mL extraction buffer containing 50 mM phosphate (pH 8.0), 10 mM Tris (pH 8.0), 500 mM NaCl, 0.1% Tween20, 0.1% NP-40, 0.1%  *β*-mercaptoethanol, 1 mM PMSF, and 1/4 of Roche Protease inhibitor cocktail MINI tablet. The crude extracts were centrifuged at 12,000 g for 10 minutes. Supernatant was transferred into a new centrifuge tube and centrifuged at 12,000 g for another 15 minutes. 10 *μ*L supernatant was mixed with 2 *μ*L 5 × SDS-PAGE loading buffer. Proteins in the extracts were separated by electrophoresis in 12% SDS-PAGE at 80 V for 1 hour and then at 120 V for another 40 minutes. Proteins in gels were transferred onto polyvinylidene difluoride PVDF membranes by Electrophoresis Cell at 60 V for 1 hour (Beijing WoDeLife Sciences Instrument Company, Beijing, China) and probed with a rabbit-anti-GFP polyclonal antibody (GenScript Co., Ltd., Nanjing, China). The polyclonal antibody was a goat-anti-rabbit IgG conjugated with alkaline phosphatase (Sigma, St. Louis, MO, USA) and used at 1 : 10000 (V/V) dilution. Proteins on the membrane were visualized by NBT-BCIP solution (Promega).

### 4.7. Analysis of* Fibrillarin*-Silenced Plants

When silenced plants developed symptoms, which usually occur at ten days after infiltration, the upper leaves of silenced plants were collected and maintained individually. Accumulation of* NbFib2* mRNA was analyzed by RT-PCR using specific primers (Table 1) designed from* NbFib2* sequence.* NbActin *mRNA (280 bp) was used as a control for the RT-PCR analysis.

## Figures and Tables

**Table 1:** The sequences, homologous recombination, and restriction sites of PCR primers.

Primer and purpose	Sequence (5′→3′)^a^	Modification
Construction for entry vector pDONR221		
*NbFib2-GF*	ggggacaagtttgtacaaaaaagcaggcttcATGGTTGCACCAACTAGAGG	Homologous recombination
*NbFib2*-GR	ggggaccactttgtacaagaaagctgggtcGGCAGCAGCCTTCTGCTTCT	Homologous recombination

Construction for VIGS vector pYL156		
*NbFib2-*VF	CGgaattcCGATGGTTGCACCAACTAGAGGTCGCG	*Eco*RI
*NbFib2-*VR	CGggatccCGTTAAATTTTCTAGGCAGCAGCCTTC	*Bam*HI

Semiquantitative RT-PCR analysis of *NbFib2* mRNA accumulation		
*NbFib2*-F	ATGGTTGCACCAACTAGAGGTCGCG	
*NbFib2*-R	GGCAGCAGCCTTCTGCTTCTTCCGGC	
*Actin*-F	ACTGATGAAGATACTCACAGA	
*Actin*-R	TGGAATTGTATGTGGTTTCAT	

^a^The lowercased letters indicate homologous recombination sequence or a restriction enzyme site.
